# PCG with biallelic CYP1B1 and CPAMD8 variants: a longitudinal case report

**DOI:** 10.3389/fopht.2026.1829921

**Published:** 2026-07-15

**Authors:** Khaled Abu-Amero, Gorka Sesma

**Affiliations:** 1 Research Department, King Khaled Eye Specialist Hospital & Research Center, Riyadh, Saudi Arabia; 2 Pediatric Ophthalmology & Strabismus Division, King Khaled Eye Specialist Hospital & Research Center, Riyadh, Saudi Arabia

**Keywords:** anterior segment dysgenesis, axial length, congenital glaucoma, CPAMD8, CYP1B1, deep sclerectomy, digenic inheritance, posterior staphyloma

## Abstract

Primary congenital glaucoma (PCG) is a rare developmental disorder caused by dysgenesis of the trabecular meshwork and anterior chamber angle. We report a five-year longitudinal case of severe bilateral PCG in a male presenting at two months of age with photophobia, tearing, corneal edema, and markedly elevated intraocular pressure (IOP). The patient required four surgical interventions, including deep sclerectomy with mitomycin C and trabeculotomy ab externo, to achieve long-term IOP control. Serial axial length measurements documented rapid buphthalmos, and ultrasound biomicroscopy revealed progressive posterior staphyloma, peripheral anterior synechiae, and iris thinning consistent with anterior segment dysgenesis (ASD). Whole exome sequencing identified compound heterozygosity: *CYP1B1* c.182G>A (p.G61E), the well-characterized Saudi founder mutation for PCG, and *CPAMD8* c.3061G>A (p.V102M), a novel ultra-rare variant predicted to be damaging. The coexistence of these two heterozygous variants in genes affecting complementary anterior segment developmental pathways supports a digenic inheritance model. To our knowledge, this is the first reported case of combined *CYP1B1* and *CPAMD8* variants in congenital glaucoma, underscoring the value of comprehensive genetic analysis in severe or atypical pediatric glaucoma presentations.

## 1 Introduction

Primary congenital glaucoma is a rare but sight-threatening developmental disorder with an estimated incidence of 1:10,000 to 1:22,000 live births, and markedly higher rates in consanguineous populations (1). It is characterized by isolated dysgenesis of the trabecular meshwork and anterior chamber angle, causing impaired aqueous humor outflow, elevated IOP, and progressive optic neuropathy. Mutations in *CYP1B1*, encoding a cytochrome P450 monooxygenase expressed in the iris, ciliary body, and trabecular meshwork, represent the most common identified molecular etiology of autosomal recessive PCG, including in Saudi Arabia, where c.182G>A (p.G61E) is the predominant founder allele (2, 3). The *CYP1B1* protein participates in retinol and arachidonic acid metabolism, modulating retinoic acid signaling critical for neural crest cell-mediated anterior segment morphogenesis (4).

*CPAMD8* encodes a complement C3/alpha-2-macroglobulin superfamily protein expressed in neural crest-derived anterior segment tissues. Biallelic *CPAMD8* loss-of-function has been established as the second most common cause of hereditary childhood open-angle glaucoma after *CYP1B1*, and has been independently associated with ASD characterized by iridocorneal angle hypoplasia and disorganized extracellular matrix (5, 6). Digenic PCG involving a heterozygous *CYP1B1* variant combined with a heterozygous ASD-gene variant is a recognized but rare mechanism, previously described with *FOXC1* and *MYOC*, producing a phenotype more severe than either single allele alone (7). The unique aspect of this case is the first reported combination of *CYP1B1* and *CPAMD8* heterozygous variants, with five years of longitudinal documentation including serial imaging, surgical records, and comprehensive molecular genetic analysis following CARE guidelines (8).

## 2 Case description

### 2.1 Patient information

A male patient was referred to a tertiary ophthalmology center at two months of age presenting with photophobia, tearing, and bilateral corneal clouding. Family history was unremarkable for ocular or systemic disease, and the patient achieved normal neurodevelopmental milestones throughout the five-year follow-up period. Pre-operative laboratory studies (complete blood count, liver function, and renal panel) were within normal limits at all time points. Written informed consent for participation and publication was obtained from the patient’s guardian in accordance with institutional guidelines (IRB approval #25006-P, King Khaled Eye Specialist Hospital & Research Center).

### 2.2 Clinical findings and surgical course

**Figure 1:**
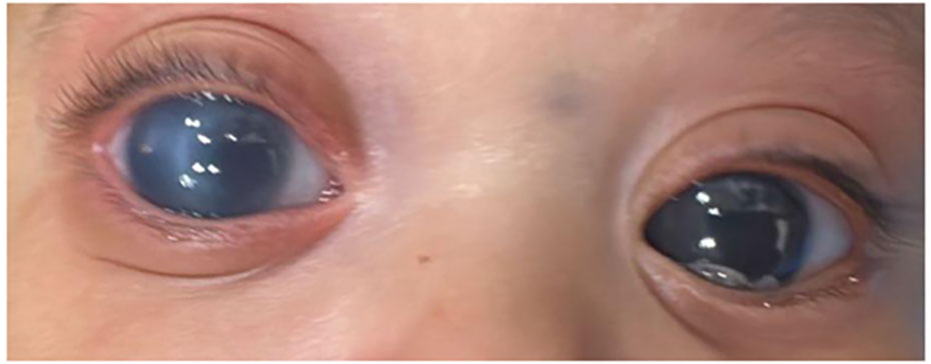
External photographs demonstrate bilateral buphthalmos with corneal edema, more pronounced in the right eye (left side of image). Marked corneal clouding and enlarged globe dimensions are evident bilaterally, consistent with elevated intraocular pressure (OD: 28 mmHg; OS: 30 mmHg at presentation).

Bilateral PCG was confirmed by examination under anesthesia (EUA). Haab’s striae and diffuse corneal edema were observed bilaterally. Pre-intubation IOP was 35 mmHg OD and 30 mmHg OS, falling to 27 mmHg OU after anesthetic induction. Horizontal corneal diameter measured 13 mm OU, with central corneal thickness (CCT) of 737 µm OD and 731 µm OS. B-scan ultrasonography demonstrated mild-to-moderate optic disc cupping and bilateral posterior staphyloma. The patient required four surgical interventions over 24 months (**Table 1**). Deep sclerectomy with mitomycin C (DS+MMC) was performed bilaterally as the initial procedure. Five months later, due to persistent IOP elevation and a corneal diameter enlarging to 15 mm with a cup-to-disc ratio (CDR) of 0.6 OD, a planned repeat DS+MMC was converted intraoperatively to 360-degree microcatheter-assisted trabeculotomy ab externo. OS required a repeat DS+MMC at 18 months (**Figure 1**).

**Table 1:** Timeline of clinical events, findings, and interventions.

Age	Event	Clinical findings	Intervention/Outcome
2 months	Initial presentation	Photophobia, tearing, bilateral corneal edema, Haab’s striae. IOP 35/30 mmHg pre-intubation. Corneal diameter 13 mm OU. CCT 737/731 µm (OD/OS). B-scan: mild-moderate optic disc cupping, bilateral posterior staphyloma.	Diagnosis of bilateral PCG. Deep sclerectomy with MMC (DS+MMC) OU.
7 months	Second surgery (OD)	IOP 35 mmHg OD. Corneal diameter 15 mm OD. CDR 0.6 OD.	Planned DS+MMC converted intraoperatively to trabeculotomy ab externo (360° microcatheter-assisted) with MMC.
18 months	Third surgery (OS)	IOP 24 mmHg OS. Corneal diameter 14 mm OS. CDR 0.5 OS.	Repeat DS+MMC OS.
8 months	AL progression	AL: 26.27/23.40 mm (OD/OS), up from 21.31/21.63 mm at 2 months. Severe axial myopia: −10.00 D OD.	Conservative; refraction monitoring initiated.
5 years	Final follow-up	IOP 14/4 mmHg (OD/OS) off medications. BCVA 20/400 OD, 20/60 OS. CDR 0.4/0.3. Anterior PAS (OD), band keratopathy with filtering bleb (OS). AL peaked at 27.84/27.56 mm.	No further surgical intervention required. Ongoing amblyopia management.

**Figure 2:**
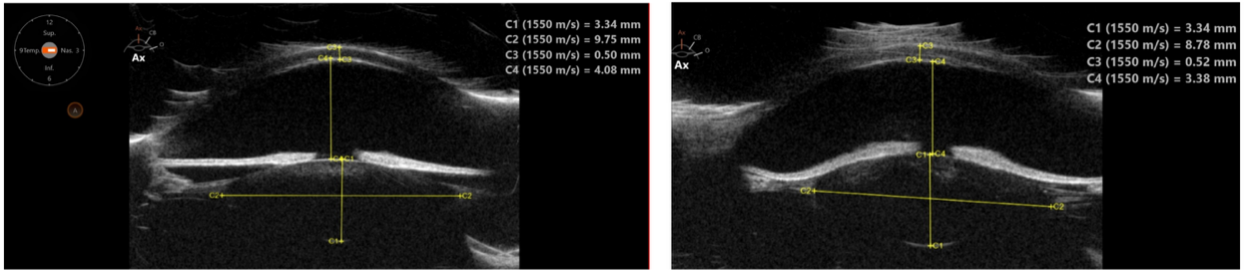
Anterior segment optical coherence tomography (AS-OCT) demonstrating characteristic CPAMD8-associated anterior segment dysgenesis features. Bilateral cross-sectional images reveal marked asymmetry between eyes: *Right eye (OD, left panel):* corneal thickness 0.50 mm, anterior chamber depth 4.08 mm, lens horizontal diameter 9.75 mm, with severe iris atrophy and concave iris configuration characteristic of neural crest-derived tissue developmental failure. *Left eye (OS, right panel):* corneal thickness 0.52 mm, anterior chamber depth 3.38 mm, lens horizontal diameter 8.78 mm, with iris atrophy present but relatively better preserved. The markedly increased anterior chamber depth in both eyes (normal 2.5-3.0 mm for age) reflect iridocorneal angle hypoplasia and trabecular meshwork dysgenesis.

At the most recent evaluation (age 5 years), IOP was 14 mmHg OD and 4 mmHg OS without medications. Best corrected visual acuity (BCVA) was 20/400 OD and 20/60 OS. CDR had partially regressed to 0.4 OD and 0.3 OS from peak values of 0.6 and 0.5. Anterior segment examination revealed bilateral Haab’s striae, iris thinning with visible lens equator on dilation, nasal posterior synechiae with pupillary irregularity (OD), and central corneal scar with band keratopathy and a diffuse superior filtering bleb (OS). Ultrasound biomicroscopy (UBM) confirmed open anterior chamber angles at all post-operative time points bilaterally, with anterior chamber depths of 4.08 mm (OD) and 3.38 mm (OS). Peripheral anterior synechiae were identified in the inferior temporal quadrant of OD at an earlier time point. CCT was 575 µm OD and 593 µm OS at final follow-up (**Figure 2**).

Axial length (AL) demonstrated rapid buphthalmic enlargement, increasing from 21.31/21.63 mm (OD/OS) at two months to 26.27/23.40 mm by eight months, and peaking at 27.84/27.56 mm. Cycloplegic refraction showed severe axial myopia at eight months (−10.00 D OD), subsequently improving to −3.50 D OD and plano OS at five years.

### 2.3 Diagnostic assessment: genetic analysis

Genomic DNA was extracted from peripheral blood. Whole exome sequencing (WES) was performed using the Roche KAPA HyperExome v2 capture system and the MGI DNBSEQ platform, with sequencing reads aligned to the hg19 reference genome (BWA v0.7.17). Mean coverage exceeded 140×, with >98% of bases covered at ≥20×. Variant calling used GATK v4.1.9.0. Copy number variants were analyzed using Exome Depth. Variant classification followed ACMG/AMP guidelines (9), and results were confirmed by Sanger sequencing.

WES identified two heterozygous missense variants in genes associated with anterior segment development (**Table 2**). The *CYP1B1* variant (c.182G>A; p.G61E; NM_000104.4, exon 2) is the established Saudi founder mutation (HGMD CM980497), with a CADD score of 23.6 and allele fraction (AF) of 61% and inherited from the father. The *CPAMD8* variant (c.3061G>A; p.V102M; NM_015692.5, exon 24) is novel, absent from HGMD and published literature, with a gnomAD allele frequency of approximately 0.0045%, CADD score of 24.5, PolyPhen-2 classified as possibly damaging, and AF of 47%. All other genes in the PCG and ASD panel were negative, including *PAX6, PITX3, FOXE3, FOXC1, LTBP2, TEK, MYOC*, and *OPTN.* This mutation was inherited from the mother.

**Table 2:** Summary of identified variants and their molecular characteristics.

Gene	Variant (cDNA)	Protein change	Zygosity (AF)	CADD score	Classification
*CYP1B1*	c.182G>A (NM_000104.4, exon 2)	p.Gly61Glu (p.G61E)	Heterozygous (AF 61%)	23.6	Pathogenic (HGMD CM980497); Saudi founder mutation
*CPAMD8*	c.3061G>A (NM_015692.5, exon 24)	p.Val102Met (p.V102M)	Heterozygous (AF 47%)	24.5	Variant of uncertain significance; novel, not in HGMD; gnomAD AF ~0.0045%; PolyPhen-2 possibly damaging

ACMG, American College of Medical Genetics and Genomics; AF, allele fraction; CADD, Combined Annotation Dependent Depletion; gnomAD, Genome Aggregation Database; HGMD, Human Gene Mutation Database.

## 3 Discussion

This case documents a five-year longitudinal course of bilateral PCG associated with digenic compound heterozygosity in *CYP1B1* (p.G61E) and *CPAMD8* (p.V102M), representing, to our knowledge, the first reported association of variants in these two genes in congenital glaucoma. The findings support a dual-pathway model: reduced *CYP1B1* activity impairs trabecular meshwork development and retinoic acid signaling, while *CPAMD8* haploinsufficiency disrupts extracellular matrix organization within the iridocorneal angle. The interaction of these two mechanistically distinct pathways likely explains the early onset, structural complexity, and surgical refractory course beyond what is typical of monoallelic *CYP1B1*-PCG.

The clinical constellation extends beyond isolated trabecular dysgenesis into two partially overlapping categories: the ASD spectrum and juvenile/childhood open-angle glaucoma (JOAG/COAG). Both *CPAMD8*-associated phenotypes have been described: Siggs et al. established *CPAMD8* as the second most common gene for hereditary childhood open-angle glaucoma after *CYP1B1*, documenting iris structural abnormalities in 81.8% of affected individuals (5). Bonet-Fernandez et al. independently linked biallelic *CPAMD8* variants to a recessive congenital glaucoma-ASD phenotype with iridocorneal angle hypoplasia and disorganized corneal extracellular matrix, validated in zebrafish morphant models (6). Peripheral anterior synechiae, progressive iris thinning, anterior lens displacement, corneal scarring, and band keratopathy in this patient are consistent with ASD-predominant features, while the open angles documented on serial UBM and long-term IOP stabilization off medications are more consistent with a chronic open-angle mechanism.

The degree of surgical complexity, four interventions within 24 months including intraoperative conversion to trabeculotomy, exceeds what is typical of isolated trabecular immaturity and supports independent contribution of *CPAMD8*-related extracellular matrix disorganization to outflow obstruction. The atypically high allele fraction of the *CYP1B1* variant (61%) warrants further investigation, as somatic mosaicism or methodological artifact cannot be excluded without additional segregation analysis. Co-harbored variants at multiple PCG-associated loci have been shown to worsen disease severity and phenotypic expressivity (10), consistent with the progressive structural findings documented here.

Visual outcomes at five years (20/400 OD; 20/60 OS) reflect the cumulative burden of early uncontrolled IOP, optic nerve damage, anisometropic amblyopia, and structural sequelae. Earlier identification of the digenic mechanism might have prompted more aggressive surgical intervention during the critical period of visual development. A limitation of this report is the absence of functional validation of the *CPAMD8* p.V102M variant and the lack of parental segregation data, which prevent definitive classification as pathogenic under current ACMG criteria.

This case illustrates three practical implications. First, heterozygous biallelic-digenic variants across two anterior segment developmental genes can produce a phenotype more severe than either allele alone. Second, UBM and serial structural imaging are essential for detecting ASD features that may signal a syndromic or digenic etiology. Third, WES-based expanded genetic panels are warranted in PCG cases where clinical severity exceeds expectations from a single mutation, as recognition of digenic inheritance may explain previously unexplained variability in glaucoma severity and guide management decisions.

The interaction of the *CYP1B1* and *CPAMD8* genes to produce the phenotype of our patient is not clear. There are several possible explanations: (i) Extracellular matrix (ECM) remodeling, whereby mutations in *CYP1B1* affect the structure of the trabecular meshwork or *CPAMD8* affect matrix turnover, leading to increased outflow resistance and higher intraocular pressure; (ii) Shared developmental signaling pathways: *CYP1B1* affects steroid signaling and *CPAMD8* could augment this through protease inhibition, leading to anterior segment dysgenesis via TGF-β, Wnt, or Notch pathways; and (iii) Oxidative stress, with partial loss of both genes potentially overwhelming cellular defense mechanisms in homozygotes. These possibilities are purely speculative at this stage and further investigations and experimental proof in animal models are needed to support these theories.

The pathogenic significance of combined *CYP1B1* and *CPAMD8* variants remains uncertain and would benefit from animal model validation. Such functional studies were beyond the scope of this work, given our laboratory’s clinical focus and lack of access to *in vivo* systems. Future studies employing transgenic or knockout models would provide direct evidence of the combined molecular effect of these variants on ocular development and disease mechanisms.

## 4 Patient perspective

The patient’s guardian reported significant anxiety during the initial diagnostic period and multiple surgical interventions. Over the five-year follow-up, the family noted relative improvement in functional vision with the left eye and expressed satisfaction with the long-term stabilization achieved without ongoing pharmacological treatment. The family emphasized the value of receiving a definitive genetic diagnosis in understanding the disease mechanism and informing counseling about recurrence risk.

## Data Availability

The raw data supporting the conclusions of this article will be made available by the authors, without undue reservation.
